# The burden of headache disorders in the adult general population of the Kingdom of Saudi Arabia: estimates from a cross-sectional population-based study including a health-care needs assessment

**DOI:** 10.1186/s10194-024-01767-6

**Published:** 2024-04-25

**Authors:** Mohammed Al Jumah, Ali M. Al Khathaami, Suleman Kojan, Andreas Husøy, Timothy J. Steiner

**Affiliations:** 1 King Abdullah International Medical Research Centre, Riyadh, Saudi Arabia; 2 King Saud Bin Abdulaziz University for Health Sciences, Riyadh, Saudi Arabia; 3 InterHealth Hospital, Riyadh, Saudi Arabia; 4 King Abdulaziz Medical City, Riyadh, Saudi Arabia; 5 NorHEAD, Department of Neuromedicine and Movement Science, Norwegian University of Science and Technology (NTNU), Trondheim, Norway; 6 Department of Neurology, University of Copenhagen, Copenhagen, Denmark; 7 Division of Brain Sciences, Imperial College London, London, UK

**Keywords:** Headache disorders, Migraine, Tension-type headache, Medication-overuse headache, Epidemiology, Burden of disease, Population-based survey, Health-care needs assessment, Eastern Mediterranean Region, Saudi Arabia, Global campaign against Headache

## Abstract

**Background:**

We have previously shown headache to be highly prevalent among adults in Saudi Arabia. Here we estimate associated symptom burden and impaired participation (impaired use of time, lost productivity and disengagement from social activity), and use these estimates to assess headache-related health-care needs in Saudi Arabia.

**Methods:**

A randomised cross-sectional survey included 2,316 adults (18–65 years) from all 13 regions of the country. It used the standardised methodology of the Global Campaign against Headache with a culturally mandated modification: engagement by cellphone using random digit-dialling rather than door-to-door visits. Enquiry used the HARDSHIP questionnaire, with diagnostic questions based on ICHD-3 beta, questions on symptom burden, enquiries into impaired participation using the HALT index and questions about activity yesterday in those reporting headache yesterday (HY). Health-care “need” was defined in terms of likelihood of benefit. We counted all those with headache on ≥ 15 days/month, with migraine on ≥ 3 days/month, or with migraine or TTH and meeting either of two criteria: a) proportion of time in ictal state (pTIS) > 3.3% *and* intensity ≥ 2 (moderate-severe); b) ≥ 3 lost days from paid work and/or household chores during 3 months.

**Results:**

For all headache, mean frequency was 4.3 days/month, mean duration 8.4 h, mean intensity 2.3 (moderate). Mean pTIS was 3.6%. Mean lost days from work were 3.9, from household chores 6.6, from social/leisure activities 2.0. Of participants reporting HY, 37.3% could do less than half their expected activity, 19.8% could do nothing. At population-level (i.e., for every adult), 2.5 workdays (potentially translating into lost GDP), 3.6 household days and 1.3 social/leisure days were lost to headache. According to HY data, mean total impaired participation (not distinguishing between work, household and social/leisure) was 6.8%. A total of 830 individuals (35.8%) fulfilled one or more of our needs assessment criteria.

**Conclusion:**

A very high symptom burden is associated with a commensurately high burden of impaired participation. The economic cost appears to be enormous. Over a third of the adult population are revealed to require headache-related health care on the basis of being likely to benefit, demanding highly efficient organization of care.

## Background

We have previously shown headache to be highly prevalent in the adult general population of the Kingdom of Saudi Arabia (KSA) [1]. Age- and gender-adjusted 1-year prevalence of any headache was 65.8%, of migraine 25.0%, of tension-type headache (TTH) 34.1%, of probable medication-overuse headache (pMOH) 2.0%, and of other headache on ≥ 15 days/month (other H15+) 2.3%. Here we present associated estimates of symptom burden and impaired participation (a term inclusive of impaired use of time, lost productivity and withdrawal or disengagement from social events and activity), and use these to assess need for headache-related health care.

KSA is a large Arab state in the Eastern Mediterranean Region (EMR), where knowledge of the burden attributable to headache remains very limited. This study, the first of its type in KSA and EMR, should, therefore, not only inform national health policy in KSA but also add to our broad understanding of headache globally, and to global burden estimates [2].

The study used the standardized methodology (with one important modification described below) developed for the Global Campaign against Headache by *Lifting The Burden* (LTB) [3, 4] a UK-registered non-governmental organization in official relations with the World Health Organization.

## Methodology

The methodology of this study has been published in detail [1]. A summary follows.

### Ethics

The Ethics Review Board of King Abdullah International Medical Research Centre approved the protocol. The study was conducted in accordance with the Declaration of Helsinki [5]. All participants gave informed consent before taking part.

Data protection legislation was complied with. Personal data were anonymised during analysis and dissemination.

### Study design

This was a cross-sectional survey of the adult population (aged 18–65 years) of KSA. It included 2,316 randomly selected individuals from all 13 regions of the country [1], well above the recommended minimum sample size of *N* = 2,000 [4] As a culturally mandated modification, we engaged with participants by cellphone using random digit-dialling rather than by unannounced door-to-door visits (the usual, and better method [4]). Trained interviewers, with nursing or other health backgrounds, interviewed eligible and willing respondents (participating proportion 86.5% [1]). The structured Headache-Attributed Restriction, Disability, Social Handicap and Impaired Participation (HARDSHIP) questionnaire [3] translated into Arabic [6] was used for the interviews.

### Headache diagnoses

The headache screening question was “Have you had a headache during the last year”. Those answering yes were asked diagnostic questions; when headaches of more than one type were reported, participants were instructed to focus in this enquiry on whichever was the most bothersome. Diagnoses were made algorithmically, with headache on ≥ 15 days/month (H15+) identified first. This was classified as pMOH when associated with reported regular use of headache medication on > 3 days/week, and otherwise as other H15+. In remaining participants, definite migraine, definite TTH, probable migraine and probable TTH were diagnosed, in that order, in accordance with ICHD criteria [7].

Further enquiry included the question “Did you have a headache yesterday?” (HY).

### Headache-attributed burden

#### Symptom burden

We estimated symptom burden at individual level in terms of frequency, duration and intensity of headache. Frequency, reported in days/month, and usual duration, reported in hours or minutes, but expressed for analysis in hours, were treated as continuous variables. Usual intensity, reported as “not bad”, “quite bad” or “very bad”, was converted into a numerical scale (1, 2 or 3). Proportion of (all) time spent in ictal state (pTIS) was calculated by multiplying headache frequency and duration and dividing by the total time available (30 days*24 hours). Since frequency was recorded in days/month, not attacks/month, duration was capped at 24 in these calculations.

Duration and intensity of HY were also recorded. pTIS for those with HY was calculated by dividing duration by 24 h.

For migraine and TTH, headache-attributed lost health was computed by multiplying pTIS by the appropriate disability weight (DW) from the Global Burden of Disease study [8].

#### Impaired participation

Two separate means of enquiry were used to estimate impaired participation at individual level.

The first used the headache-attributed lost time (HALT-90) questionnaire [9]. Its five questions distinguished between impaired participation resulting in lost productivity, separately from paid (questions 1 and 2) and household work (questions 3 and 4), and lost social or leisure time (question 5) over the preceding 3 months [9]. For the former, days of nothing or less than half achieved were totalled in accordance with accepted methodology [9] (counterbalanced by interpreting more than half achieved as everything achieved). For the latter, we counted reported days of missed social or leisure activity or occasions.

The second enquiry was into impaired participation yesterday among those with HY, without distinguishing between paid or household work and social activity. Response options were everything, more than half, less than half or nothing achieved yesterday of whatever had been planned. Again we took less than half as nothing achieved, and, in counterbalance, more than half as everything achieved.

#### Population-level estimates

pTIS, lost health and impaired participation at population level were calculated by factoring in prevalence estimates and adjusting for age and gender.

We were also able to make population-level estimates of pTIS and lost productivity based on HY, factoring in 1-day prevalence of any headache and again adjusting for age and gender. We chose to do this only for all headache, recognizing that ICHD criteria do not permit diagnosis of individual headache episodes [7]. We could infer the diagnosis of HY whenever headache was of only one type, or HY was reported to be of the same type as the (diagnosed) most bothersome headache, but our inability to do so in all cases precluded estimation of 1-day prevalence of each type.

### Health-care needs assessment

We defined “need” for health care in terms of numbers likely to benefit from health care, setting opinion-based criteria for bothersomeness, likelihood of negative impacts on participation and quality of life, and expectation of need for prescription medication (including preventative). Accordingly, we counted all participants with H15+, all those with migraine on ≥ 3 days/month, and those with migraine or TTH who met one or both of the following criteria: a) pTIS > 3.3% *and* intensity ≥ 2 (moderate-severe); b) ≥ 3 lost workdays and/or lost household days during the preceding 3 months. These counts were adjusted for age- and gender-composition of the sample to yield population estimates.

### Statistics

Means, standard deviation (SDs), standard errors of the mean (SEMs) and medians were used to describe continuous variables. Group-differences were examined using ANOVA for continuous variables and chi-squared tests for categorical variables.

Statistical analyses were conducted using SPSS version 28 for statistical analysis (SPSS, INC, Chicago, IL). The threshold for significance was set at *p* < 0.05.

## Results

Of the 2,316 participants (males 62.3%; mean age 32.2 ± 10.7 years) in the original study (with a non-participating proportion of 13.5% [1]), 1,789 reported headache of any type, of whom 663 were diagnosed with migraine (definite or probable), 994 with TTH (definite or probable), 41 with pMOH and 48 with other H15+ [1]. The age- and gender-adjusted 1-year prevalences were 25.0% (95% CI: 23.2–26.8), 34.1% (32.2–36.0), 2.0% (1.5–2.7) and 2.3% (1.7-3.0) respectively (total for these headache types: 63.4%). HY was reported by 254 participants; adjusted 1-day prevalence of any headache was 11.5% [1]. These findings and estimates have been reported previously [1], but are recorded here since they are used in the analyses.

### Symptom burden

Table 1 shows the headache-attributed symptom burden. For all headache, mean frequency was 4.3 days per month, mean duration 8.4 h and mean intensity 2.3 (moderate). Mean pTIS was 3.6%. Females reported significantly higher frequency than males (5.1 vs. 3.7 days per month), with accordingly higher pTIS (4.8% vs. 2.7%) (Table 1).

For migraine, mean frequency was 3.5 days/month, mean duration 12.1 h and mean intensity 2.4 (moderate-to-severe). Mean pTIS was 3.5%. Mean estimated lost health attributed to migraine was 1.5%. There were no gender-related differences in symptom burden of migraine. TTH was rather less frequent (mean 3.0 days/month), of shorter duration (mean 3.8 h) and of rather less intensity (mean 2.0: moderate). Mean pTIS was 1.3%. Mean estimated lost health attributed to TTH was 0.0% (i.e., below the limits of estimation). Frequency was slightly but significantly higher in females than males (3.3 vs. 2.9 days/month) (Table 1).

pMOH was present on more days than not (mean 19.7 days/month), as by definition it must be, with a mean duration of 6.1 h and a mean intensity of 2.6 (moderate-to-severe). Mean pTIS was 17.1%. There were no significant differences between males and females.

Other H15 + was present on a mean of 15.9 days/month, with a mean duration of 26.6 h and a mean intensity of 2.4 (moderate-to-severe). Mean pTIS was 21.4%. Mean intensity was higher among females than males (2.6 vs. 2.2) (Table 1).

**Table 1:** Symptom burden by headache type and gender

	Overall	Male	Female	Male vs. female
	Mean±SEM, median			
**Frequency** (days/month)				
Any headache	4.3±0.1, 3.0	3.7±0.1, 2.0	5.1±0.2, 3.0	*p* < 0.001
pMOH	19.7±0.7, 20.0	18.1±1.0, 15.0	20.5±0.8, 20.0	*p* = 0.08
Other H15+	15.9±1.1, 15.0	14.9±1.7, 15.0	16.6±1.5, 15.0	*p* = 0.45
Migraine	3.5±0.1, 3.0	3.4±0.2, 2.0	3.7±0.2, 3.0	*p* = 0.21
TTH	3.0±0.1, 2.0	2.9±0.1, 2.0	3.3±0.2, 2.0	*p* = 0.04
**Duration** (hours)				
Any headache	8.4±0.7, 3.0	7.9±1.0, 2.0	9.1±0.7, 3.0	*p* = 0.37
pMOH	6.1±0.7, 3.0	5.5±0.9, 5.0	6.4±1.0, 3.0	*p* = 0.59
Other H15+	26.6±18.3, 4.0	49.0±41.9, 3.0	9.2±1.7, 5.0	*p* = 0.29
Migraine	12.1±0.8, 4.0	11.4±1.1, 4.0	12.8±1.2, 4.0	*p* = 0.42
TTH	3.8±0.4, 2.0	3.5±0.4, 2.0	4.4±1.0, 2.0	*p* = 0.27
**Intensity** (mild, moderate, severe; equated to 1, 2, 3)				
Any headache	119-836-490 (mean = 2.3)	77-511-253 (mean = 2.2)	42-325-237 (mean = 2.3)	*p* = 0.001
pMOH	0-23-39 (mean = 2.6)	0-8-11 (mean = 2.6)	0-15-28 (mean = 2.7)	*p* = 0.59
Other H15+	1-21-16 (mean = 2.4)	0-15-3 (mean = 2.2)	1-7-13 (mean = 2.6)	*p* = 0.01
Migraine	27-355-318 (mean = 2.4)	13-199-166 (mean = 2.4)	14-156-152 (mean = 2.4)	*p* = 0.50
TTH	81-417-100 (mean = 2.0)	56-275-64 (mean = 2.0)	25-142-36(mean = 2.1)	*p* = 0.77
**Proportion of time in ictal state** (%)				
Any headache	3.6±0.2, 1.1	2.7±0.2, 0.8	4.8±0.4, 1.4	*p* < 0.001
pMOH	17.1±2.3, 10.0	13.8±2.2, 12.5	18.5±3.2, 8.3	*p* = 0.35
Other H15+	21.4±4.2, 8.3	16.7±6.0, 6.3	25.0±5.9, 11.1	*p* = 0.34
Migraine	3.5±0.2, 1.6	3.2±0.2, 1.4	3.9±0.3, 1.6	*p* = 0.08
TTH	1.3±0.1, 0.5	1.2±0.1, 0.5	1.5±0.2, 0.5	*p* = 0.11
**Headache-attributed lost health** (%)				
Migraine	1.5±0.1, 0.7	1.4±0.1, 0.6	1.7±0.1, 0.7	*p* = 0.08
TTH	0.0±0.0, 0.0	0.0±0.0, 0.0	0.1±0.0, 0.0	*p* = 0.11

pMOH: probable medication-overuse headache; H15+: headache on ≥ 15 days/month; TTH: tension-type headache

### Impaired participation

**Fig. 1:**
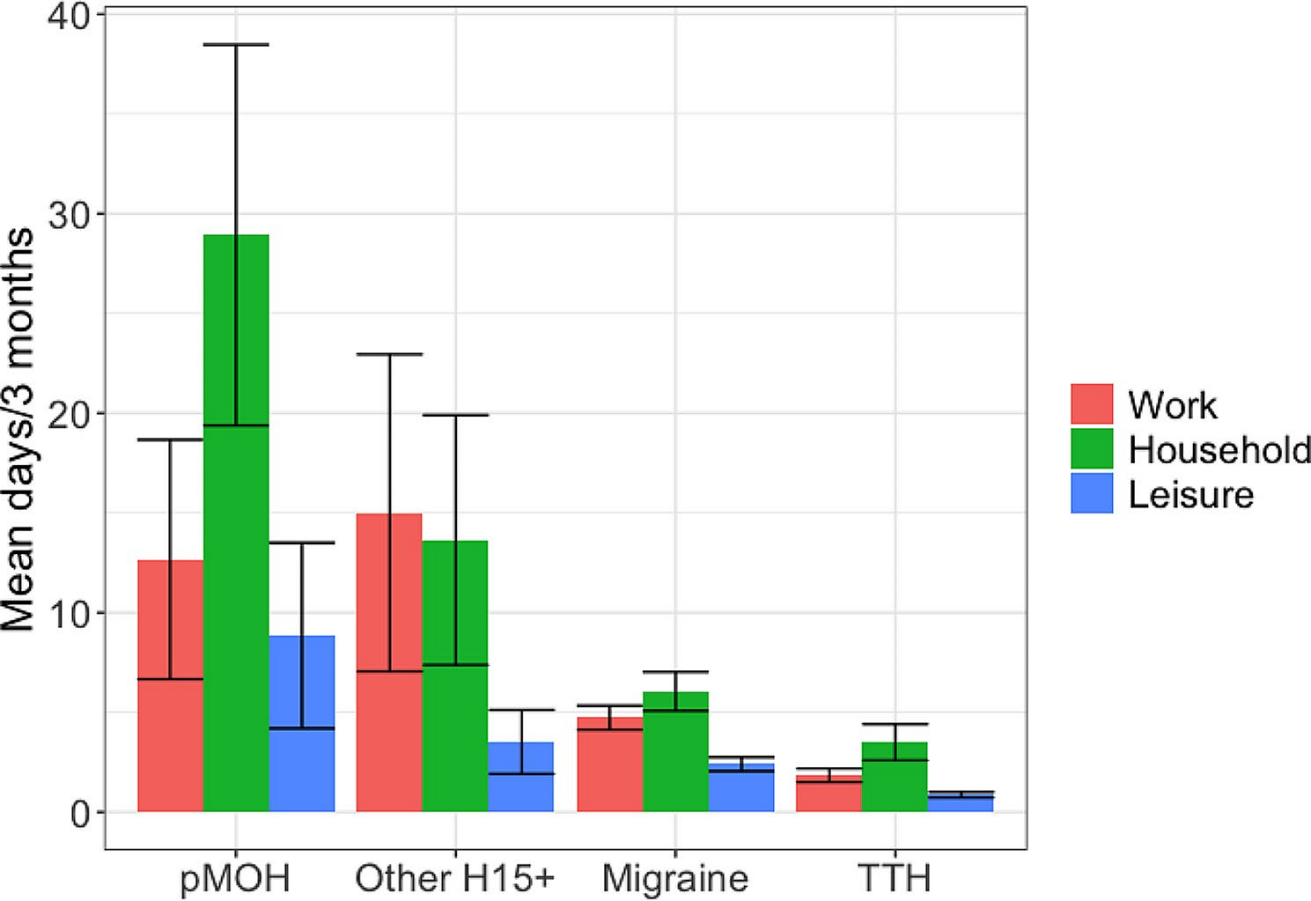
Headache-attributed impaired participation by headache type (error bars = 95% confidence interval)

Headache type had a highly significant (*p* < 0.001) influence on lost productivity from both paid and household work (Table 2; Fig. 1). Participants with pMOH reported losing 12.7 workdays and 28.9 household days in the preceding 3 months; those with other H15 + reported losing 15.0 work and 13.6 household days over the same period. Those with migraine reported 4.7 and 6.0 lost work and household days; those with TTH reported 1.8 and 3.5 lost work and household days. Overall (for any headache), females reported higher losses than males from both paid (5.7 vs. 3.3 days) and household work (7.1 vs. 3.9 days) (Table 2), but there were no significant gender-related differences for any of the headache types.

**Table 2:** Headache-attributed impaired participation from HALT data by headache type and gender

Headache type	Overall	Male	Female	Male vs. female
	Mean±SEM, median			
**Lost workdays in preceding 3 months**				
Any headache	3.9±0.2, 2.0	3.3±0.2, 2.0	5.7±0.7, 3.0	*p* < 0.001
pMOH	12.7±2.9, 10.0	8.3±1.8, 8.5	17.4±5.6, 12.0	*p* = 0.12
Other H15+	15.0±3.8, 10.0	14.5±4.8, 6.0	15.8±6.5, 10.0	*p* = 0.87
Migraine	4.7±0.3, 3.0	4.6±0.3, 3.0	5.1±0.6, 3.0	*p* = 0.49
TTH	1.8±0.2, 1.0	1.8±0.2, 0.0	2.4±0.6, 1.0	*p* = 0.19
	*p* < 0.001			
**Lost household days in preceding 3 months**				
Any headache	6.6±0.5, 3.0	3.9±0.7, 2.0	7.1±0.6, 3.0	*p* = 0.02
pMOH	28.9±4.6, 20.0	21.0±4.0, 21.0	29.6±5.0, 20.0	*p* = 0.64
Other H15+	13.6±2.8, 12.0	30.0±0.0, 30.0	12.0±2.5, 10.5	*p* = 0.06
Migraine	6.1±0.5, 4.0	4.2±0.7, 3.0	6.4±0.6, 4.0	*p* = 0.09
TTH	3.5±0.5, 2.0	1.8±0.6, 0.0	3.9±0.5, 2.0	*p* = 0.07
	*p* < 0.001			
**Lost social or leisure days in preceding 3 months**				
Any headache	2.0±0.1, 1.0	1.4±0.1, 0.0	2.7±0.3, 1.0	*p* < 0.001
pMOH	8.9±2.3, 4.0	2.3±0.7, 1.0	10.9±2.9, 5.5	*p* = 0.12
Other H15+	3.5±0.8, 2.0	2.9±1.0, 2.0	3.8±1.1, 2.5	*p* = 0.58
Migraine	2.4±0.2, 1.0	2.2±0.2, 1.0	2.5±0.3, 1.0	*p* = 0.25
TTH	0.9±0.1, 0.0	0.7±0.1, 0.0	1.1±0.1, 0.0	*p* = 0.01
	*p* < 0.001			

pMOH: probable medication-overuse headache; H15+: headache on ≥ 15 days/month; SEM: standard error of the mean

**Table 3:** Symptom burden and impaired participation attributed to headache yesterday

Burden measure	Overall	Male	Female	Male vs. female
**Duration** (hours)				
(mean±SEM, median)	5.2±0.4, 3.0	4.5±0.5, 3.0	5.8±0.5, 3.0	p = 0.06
**Intensity** n (%)				
mild	30 (11.8)	15 (11.8)	15 (11.8)	
moderate	155 (61.0)	83 (65.3)	72 (56.7)	
severe	69 (27.2)	29 (22.8)	40 (31.5)	
mean*	2.2	2.1	2.2	p = 0.28
**What done** n (%)				
everything	66 (26.2)	43 (34.1)	23 (18.3)	
more than half	42 (16.7)	18 (14.3)	24 (19.0)	
less than half	94 (37.3)	46 (36.5)	48 (38.1)	
nothing	50 (19.8)	19 (15.1)	31 (24.6)	p = 0.02

* Equating to 1, 2, 3, and treating as though continuous data

Losses from social or leisure time were smaller than those from paid or household work: males with (any) headache lost 1.4 and females 2.7 days during the preceding 3 months (*p* < 0.001). Again, headache type had a significant (*p* < 0.001) influence: in particular, pMOH was associated with 8.9 lost social or leisure days in the preceding 3 months, TTH with only 0.9 days. There were no significant gender-related differences for any of the headache types.

### Headache yesterday

Symptom burden and impaired participation attributed to HY (*N* = 254) are shown in Table 3. The mean duration of HY was 5.2 h, equating to a pTIS of 21.7%, and mean intensity was 2.2 (moderate). Neither was significantly different between males and females.

Of all participants reporting HY and responding with regard to impaired participation (*n* = 252), 26.2% could do everything as normal, 16.7% could do more than half, 37.3% could do less than half and 19.8% could do nothing (Table 3). Females reported greater impairment of participation yesterday than males (*p* = 0.02).

### Population-level estimates

At population level, the estimated pTIS with (any) headache was 2.5% based on 30-day recall and 2.7% based on HY (Table 4). More time was spent with migraine (1.1%) than pMOH (0.6%), other H15+ (0.4%) or TTH (0.3%).

Mean population-level lost health attributed to migraine was 0.5% and attributed to TTH was 0.0% (i.e., below the limits of estimation).

Estimates of impaired participation at population-level are also shown in Table 4. According to HALT data (over 3 months), 2.5 workdays, 3.6 household days and 1.3 social or leisure days were lost due to (any) headache. Migraine had the biggest impacts on productivity in both domains (1.4 lost workdays, 1.9 lost household days) and on social or leisure time (0.8 lost days), double those of TTH, with pMOH and other H15 + some way behind (Table 4). According to HY data, total impaired participation was estimated to be 6.8%.

### Health-care needs assessment

A total of 830 individuals (35.8% of our sample) fulfilled one or more of our needs assessment criteria (Table 5). Adjusted for age and gender, the proportion of the adult population of KSA with headache likely to benefit from health care stayed the same (35.8% [95% CI: 33.9–37.8]). The age- and gender adjusted proportions with migraine or TTH likely to benefit from health care were 22.7% [21.0-24.5] and 8.2% [7.1–9.4] respectively.

**Table 4:** Proportion of time in ictal state and impaired participation at population level by headache type and by timeframe of enquiry (adjusted for age and gender)

Headache type	Estimated pTIS (%)		Estimated impaired participation			
	According to 1-year prevalence* and reported average frequency and duration	According to prevalence* and duration of headache yesterday	According to HALT data (lost days/3 months)			According to headache yesterday
			Lost productivity		Lost social or leisure	Total impaired participation (%)
			Paid work	Household work		
Any headache	2.5	2.7	2.5	3.6	1.3	6.8
Migraine	1.0		1.4	1.9	0.8	
TTH	0.5		0.7	0.9	0.6	
pMOH	0.3		0.2	0.3	0.1	
Other H15+	0.4		0.2	0.2	0.1	

pTIS: proportion of time in ictal state; HALT: headache-attributed lost time; TTH: tension type headache; pMOH: probable medication-overuse headache; H15+: headache on ≥ 15 days/month. *Prevalence estimates are from [1], and are iterated above

**Table 5:** Health-care needs assessment

Criterion fulfilled		Proportion of sample		Estimated proportion of adult population*
		n	%	% [95% CI]
1	Headache on ≥ 15 days/month	100	4.3	4.3 [3.5–5.2]
2	Migraine on ≥ 3 days/month	356	15.4	15.2 [13.8–16.7]
3	Migraine and pTIS > 3.3% and moderate-severe intensity	191^1^	8.2	8.0 [6.9–9.2]
4	Migraine and lost work and/or household days/3 months ≥ 3	370^2^	16.0	14.8 [13.4–16.3]
5	TTH and pTIS > 3.3% and moderate-severe intensity	38	1.6	2.0 [1.5–2.7]
6	TTH and lost work and/or household days/3 months ≥ 3	157^3^	6.8	6.9 [5.9-8.0]
One or more of criteria 1–6		830	35.8	35.8 [33.9–37.8]

*Age- and gender-corrected; ^1^of whom 150 also fulfilled criterion 2; ^2^of whom 192 also fulfilled criterion 2, 119 also fulfilled criterion 3, and 94 also fulfilled criteria 2 and 3; ^3^of whom 15 also fulfilled criterion 5

## Discussion

Having previously shown headache disorders to be highly prevalent in KSA (with estimates for migraine and TTH both higher than global averages [1, 10, 11]), here we demonstrate commensurately high levels of attributed burden.

With regard to symptom burden, for all headache, mean frequency was 4.3 days/month, mean duration 8.4 h and mean intensity 2.3 (moderate). Thus, participants reporting any headache in the preceding year spent, on average, 3.6% of all their time with headache (3.5% for those with migraine, 1.3% for those with TTH and, of course, much more for those with H15+ [17.1 and 21.4% for pMOH and other H15 + respectively]). By factoring in prevalence, we estimated 2.5–2.7% of all time among the total adult population of KSA was spent with headache (1.0% with migraine, 0.5% with TTH, 0.3% with pMOH and 0.4% with other H15+).

From the DWs supplied by GBD [8], estimated mean population-level lost health was 0.4% attributed to migraine and below the limits of estimation for TTH. These low values were belied by population-level lost productivity estimates: for migraine 1.4 workdays and 1.9 household days over 3 months; for TTH 0.7 workdays and 0.9 household days over 3 months. These findings suggest a disconnect between lost health and lost productivity, with either or both of two explanations: (a) headache-attributed lost health is not the sole cause of headache-attributed lost productivity; (b) the former is greatly underestimated. Estimates of lost health take account only of pTIS, itself defined by duration of headache, and so ignore any lost health that might be attributable to premonitory [12, 13] or postdromal symptoms [14] or occasioned interictally [15].

Lost productivity from paid work at population-level attributed to all headache was 2.5 days/3 months. If this translates into lost gross domestic product (GDP), then headache has a huge impact on the economy of KSA. Estimated lost productivity from household work was even higher (3.6 days/3 months). “Household work” means the chores necessary for daily life, and losses here must also have economic impact. So, arguably, does withdrawal from social activity (1.3 days/3 months), both because this is expected to impair wellbeing, and therefore function on a broad scope, and because it reduces consumption.

With regard to headache type, migraine was responsible for more lost productivity at individual level than TTH, but the impacts of pMOH and other H15 + were greater by far (respectively, 12.7 and 15.0 workdays and 28.9 and 13.6 household days lost over 3 months). Nevertheless, pMOH and other H15 + had smaller impacts at population level than migraine or TTH because of their much lower prevalences. In a patriarchal society, large gender-related differences might be expected in the balance of work and household days lost, but these were not seen. In general, all gender differences in burden were small.

While HALT data provide for estimates of impaired participation in three domains, HY data offer only an overall estimate. We refrain from the temptation to make comparisons. Were we to generate summary measures from HALT data, we might, from Table 4, arrive at a sum for all headache of 7.4 (2.5 + 3.6 + 1.3) days lost in 3 months, and a proportion of 8.2% (7.4/90) to compare with 6.8% from HY data. The fundamental problem is the unknowable denominator for HALT: how many days in 3 months were workdays, household days or days set aside for social and leisure activities? Furthermore, a participant might count one day as lost in more than one of these domains if, for example, both a day of work and an evening of leisure had been planned. We might expect estimates within the same order of magnitude, as they were here, but HALT and HY data each provide unique and complementary insights into impaired participation.

Finally, we come to the needs assessment, and its formidable finding: an estimated 35.8% of the adult population of KSA need (i.e., would be expected to benefit from) professional headache care. Policy makers who might balk at providing health care for so many should be aware of the lost-productivity findings in Table 4. Population-level loss of 2.5 days/3 months from income-generating work translates into 10 days/year. It might be expected that this needs estimate would be sensitive to the criteria we set to define “need”. Inclusion of all those with H15+ (pMOH or other) seems uncontroversial, as does inclusion of all with ≥ 3 migraine days/month (a commonly applied threshold for preventative drugs [16]). The other criteria, which lead almost to a doubling of the estimated need (Table 5), may be more questionable: those with migraine or TTH, with pTIS > 3.3% (equivalent to one full day per month) *and* moderate or greater intensity; those with migraine or TTH losing ≥ 3 work and/or household days/3 months. If both were arbitrarily increased by 100% (pTIS > 6.6% [equivalent to two full days per month], and/or ≥ 6 lost work and/or household days/3 months), the estimated proportion with need would fall to a slightly more manageable 27.1%. Even in a high-income country, some prioritization of this sort is probably necessary.

### Strengths and limitations

Strengths of the study were the use of standardized methodology (with one important modification) and questionnaire, the large and adequate sample size, and the low non-participating proportion (13.5%).

The modification has already been described as an important study limitation [1]: sampling was by random-digit dialling of cellphones rather than house-to-house visits. This was a culturally necessary but suboptimal method of engagement with participants [1], even though ownership of cellphones was almost universal in KSA at the time of the study [17]. The imperfect match of gender and age composition in the sample with that of the national adult population [1] was a probable consequence, but corrections for age and gender were made when estimating population-level burden, and in the needs assessment.

A general limitation of population-based surveys is their dependence on participants’ recall. A strength was inclusion of enquiry into symptom burden and impaired participation on the day preceding the interview, eliminating recall error. Of course, 1-day prevalence is much lower than 1-year prevalence, reducing precision, but our estimates based on HALT and HY data to a large degree corroborated each other.

## Conclusion

With regard to our aim of informing national health policy in KSA, this study has demonstrated a high headache-attributed symptom burden: 63.4% of the adult population spend, on average, 3.6% of all their time with headache of at least moderate severity. There is a commensurately high burden of impaired participation. The economic cost appears to be enormous: headache is responsible for an average of 2.5 workdays lost every 3 months per person in the adult population of KSA (with or without headache), and for even higher losses (3.6 days) from work that is non-paid but necessary nonetheless to maintain everyday life. These are clear and urgent targets for health care. The very large numbers revealed by this study to require it (on the basis of being likely to benefit) demand highly efficient organization of care, such as we have proposed in structured headache services [18, 19].

This study also adds to our understanding of headache and its consequences globally, since it is the first of its kind to be reported not just from KSA but from the Eastern Mediterranean Region.

## Data Availability

Full electronic data are held securely at King Abdullah International Medical Research Centre, and the analytical subset at Norwegian University of Science and Technology (NTNU), Trondheim, Norway. When analyses are completed, the latter will be available on request for academic purposes.

## Abbreviations

CI: Confidence interval
DW: Disability weight
GBD: Global Burden of Disease
GDP: Gross domestic product
HARDSHIP: Headache-Attributed Restriction, Disability, Social Handicap and Impaired Participation questionnaire
EMR: Eastern Mediterranean Region
KSA: Kingdom of Saudi Arabia
ICHD: International Classification of Headache Disorders
HALT: Headache-attributed lost time
HY: Headache yesterday
LTB: *Lifting The Burden*
MOH: Medication-overuse headache
pMOH: Probable MOH
SD: Standard deviation
SEM: Standard error of the mean
TTH: Tension-type headache
